# Data on the evolution of curing characteristics and properties during the room-temperature annealing process in SSBR/BR gums and SSBR/BR/SiO_2_ composites

**DOI:** 10.1016/j.dib.2019.104660

**Published:** 2019-10-16

**Authors:** Xinping Zhang, Lei Cai, Chuanwei Wang, Aihua He

**Affiliations:** Shandong Provincial Key Laboratory of Olefin Catalysis and Polymerization, Key Laboratory of Rubber-Plastics, Ministry of Education/Shandong Provincial Key Laboratory of Rubber-Plastics, School of Polymer Science and Engineering, Qingdao University of Science and Technology, Qingdao 266042, China

**Keywords:** Polymer composites, Mechanical properties, Annealing, Curing characteristics, Filler dispersion

## Abstract

The present article contains the data of tensile stress-strain curves, crosslinking characteristics curves, filler dispersion images and dynamic mechanical properties of SSBR/BR blends and SSBR/BR/SiO_2_ composites during room-temperature annealing. The data in this article aims to accurately describe the evolution of the network structures and physical mechanical properties of rubber composites during annealing process. Tensile stress-strain curves of un-vulcanized rubber gums and compounds were obtained by an electrical tensile tester with a speed of 100 mm/min. The crosslinking characteristics of the un-vulcanized rubber blends or composites after different annealing time were measured by a rotor-free vulcameter at 150 °C. The macroscopic filler dispersion of the filled vulcanizates was analyzed on a smooth cross-section of cut rubber. The dynamic mechanical properties of vulcanized SSBR/BR blends and SSBR/BR/SiO_2_ composites were investigated by a dynamic mechanical thermal analyser with different temperature ranges and strains. These findings may serve as references for the scientific processing of green tire materials in automotive industry, and this article is related to our research article entitled “Effect of room-temperature annealing on structures and properties of SSBR/BR blends and SSBR/BR/SiO_2_ composites” (Xinping Zhang et al., 2019).

**Table:** Specifications Table

Subject	Materials Science
Specific subject area	Polymeric materials applied in the green tire treads
Type of data	Image Figure
How data were acquired	The tensile properties of un-vulcanized rubber compound were measured by with a Zwick/Roell Z005 electrical tensile tester (Zwick, Germany) with a speed of 100 mm/min according to ISO 9026-2007. The crosslinking characteristics were determined at 150 °C with RHEOMETER MDR 2000 (Alpha Technologies Co. the US). The macroscopic filler dispersion analysis was tested using disper GRADERTM αview (Alpha Technologies Co. the US) according to ISO 11345-2006. Dynamic mechanical analysis (DMA) was performed on a tension mode by Q800 dynamic mechanical thermal analyser (TA, United States). Each sample was 10 mm in length, 4 mm in width, and 2 mm in thickness. Testing conditions for obtaining the value of tan δ at 0 °C were as follows: temperature sweep was conducted at a frequency of 10 Hz from −80 °C to 100 °C with a heating rate of 3 °C/min and a strain amplitude of 0.1%. Testing conditions for obtaining the value of tan δ at 60 °C were as follows: temperature sweep was conducted at a frequency of 10 Hz from 20 °C to 100 °C with a heating rate of 3 °C/min and a strain amplitude of 5.0%.
Data format	Raw Analyzed
Parameters for data collection	The annealing temperature, annealing time and the settings of instruments and experimental parameters.
Description of data collection	The annealing temperature were controlled at 23 °C, and annealing time was set as 0 day, 2 days, 4 days, and 7 days. The vulcanizates were cured at 150 °C under 10 MPa. The changes of the structures and performances were monitored and correlated with the annealing time.
Data source location	Shandong Provincial Key Laboratory of Olefin Catalysis and Polymerization, Key Laboratory of Rubber-Plastics, Ministry of Education/Shandong Provincial Key Laboratory of Rubber-Plastics, School of Polymer Science and Engineering, Qingdao University of Science and Technology, Qingdao 266042, China
Data accessibility	With the article
Related research article	Xinping Zhang, Lei Cai, Chuanwei Wang, Aihua He, Effect of room-temperature annealing on structures and properties of SSBR/BR blends and SSBR/BR/SiO_2_ composites, Composites Science and Technology [1] DOI

**Table:** 

**Value of the Data** • The data can be used to understand the effects of room-temperature annealing process on the evolution of network structures and properties of rubber composites. • Researchers in the field of the design and development of high-performance polymer composites (especially, the composites used in green tires industry). • These findings could promote researchers' understanding towards the evolution of materials during annealing. • The data can provide the optimal properties and network structures of the rubber composites during the annealing process, hence the optimal annealing time can be selected to achieve adequate material performances. • The data has potential value for the modern tire industry and future polymeric rubber materials research.

## 1 Data

The investigated data shown in this report demonstrate the evolution of the network structures and properties of SSBR/BR blends and SSBR/BR/SiO_2_ composites during the room-temperature annealing. The stress-strain curves of un-vulcanized rubber gums and compounds during room-temperature annealing is shown in Fig. 2. Then the curing characteristic curves of rubber gums and compounds is displayed in Fig. 3. The data in Fig. 4 and Fig. 5 show the macroscopic filler dispersion (detecting large filler aggregates with dimension in the range of 3–57 μm) in filled SSBR/BR/SiO_2_ vulcanizates during room-temperature annealing. Additionally, the dynamic mechanical properties of the SiO_2_ filled SSBR/BR vulcanizates are shown Fig. 6.

**Fig. 1:**
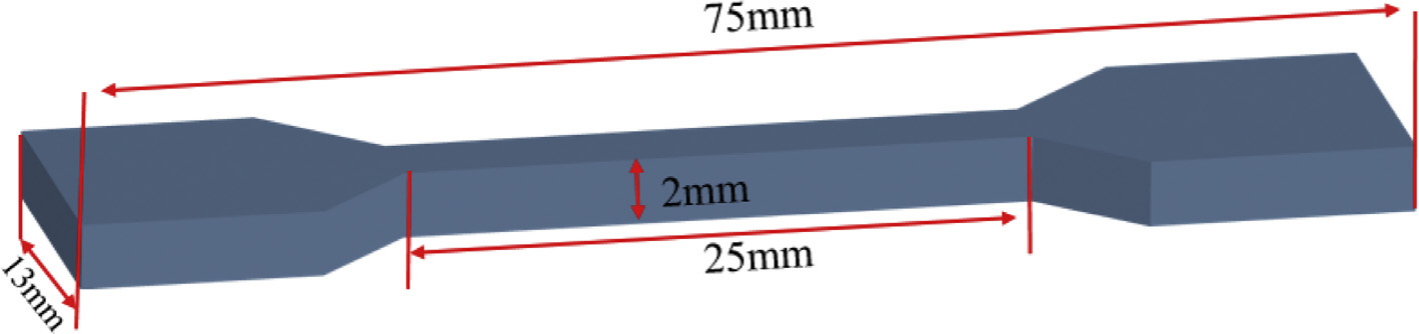
Standard dumbbell-shaped specimen (all the dimensions are in mm).

**Fig. 2:**
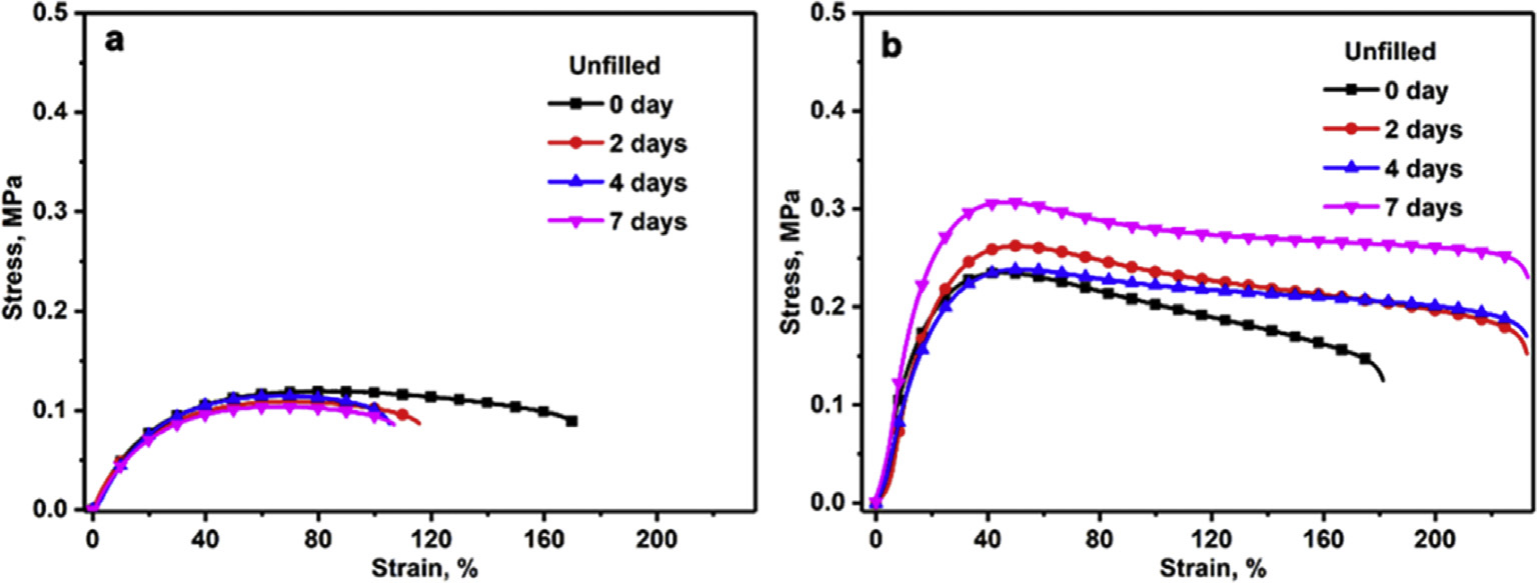
Stress-strain curves of the un-vulcanized unfilled rubber gums and filled rubber compounds after annealing for different periods.

**Fig. 3:**
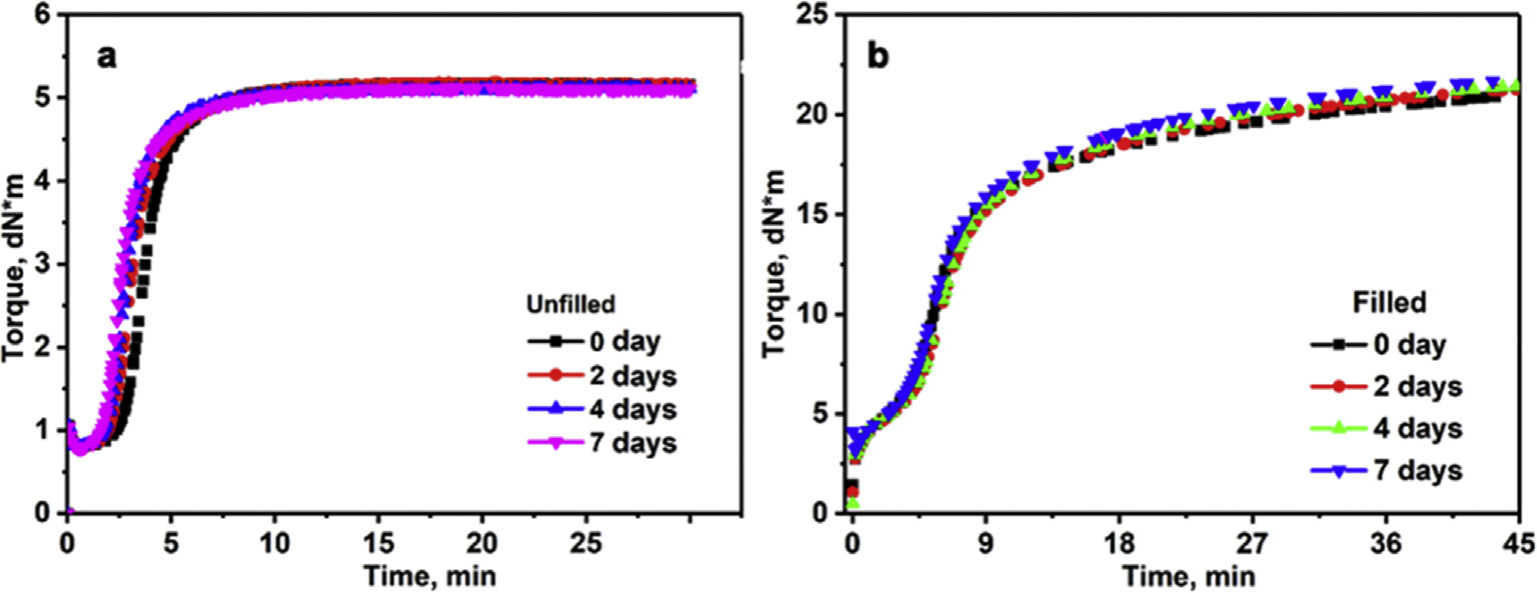
Crosslinking characteristics of the unfilled rubber gums and filled rubber compounds after annealing for different periods.

**Fig. 4:**
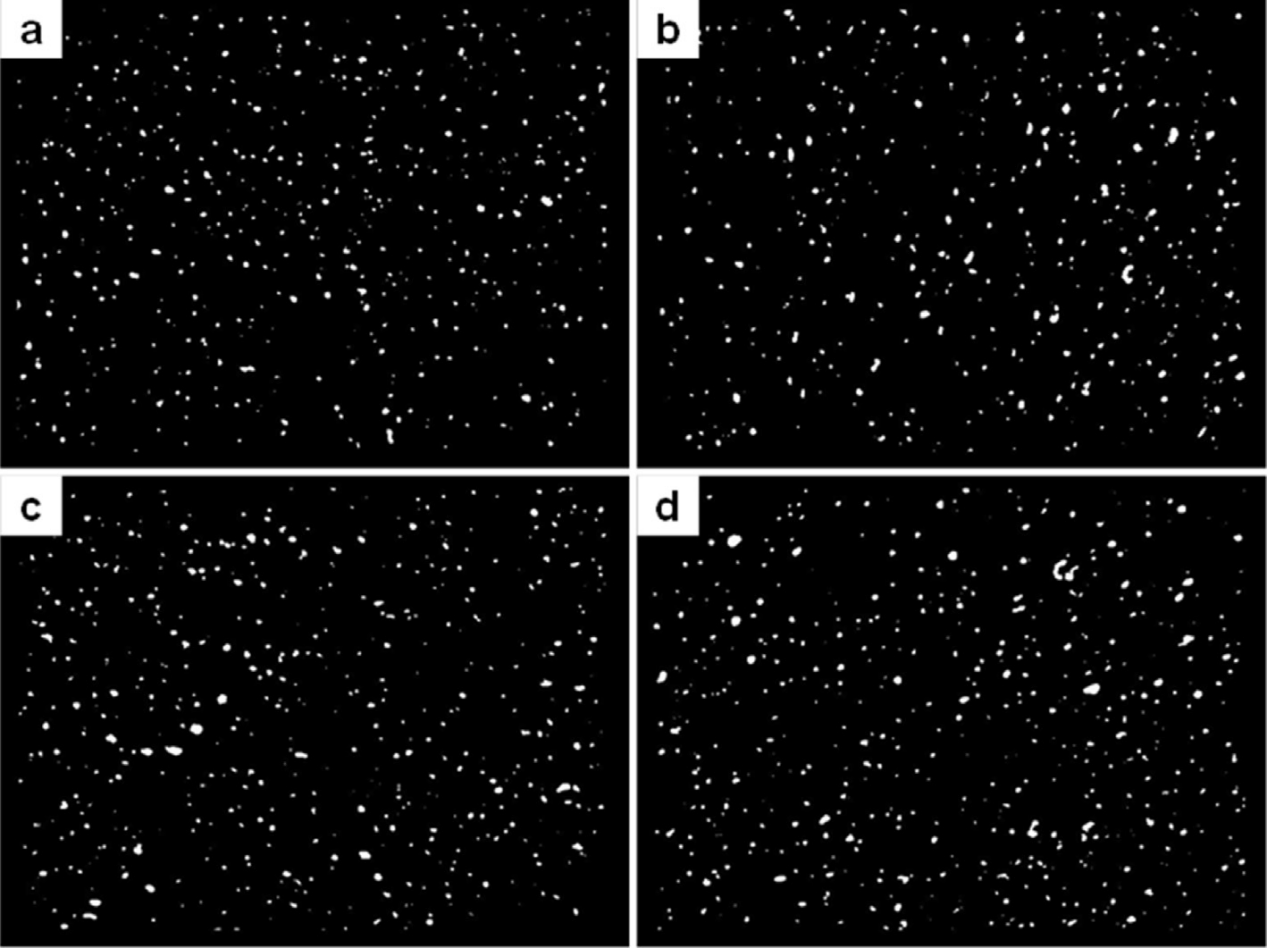
The macroscopic filler dispersion images of filled SSBR/BR vulcanizates after annealing for a) 0 day, b) 2 days, c) 4 days, and d) 7 days.

**Fig. 5:**
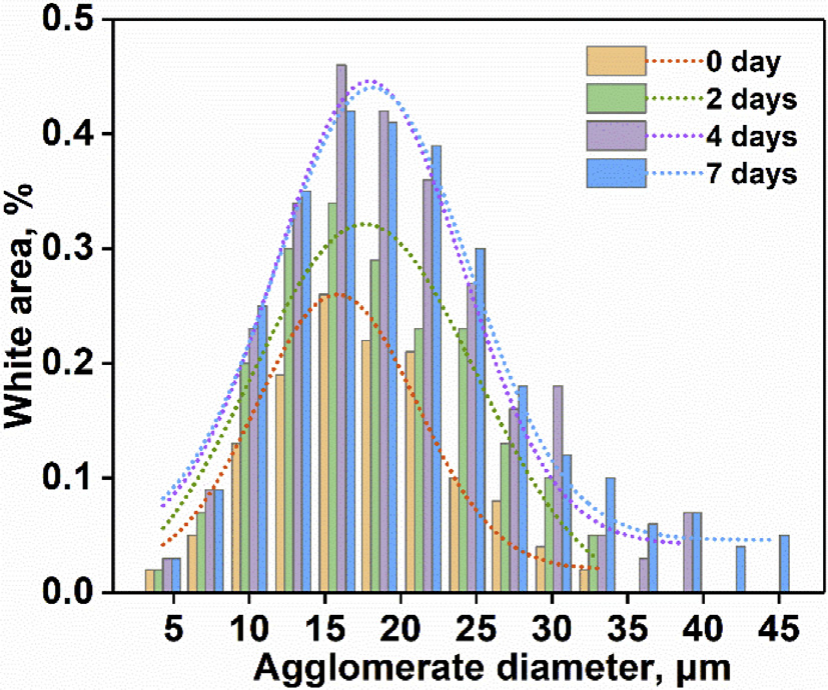
2D histogram and Gauss fitting of filler agglomerate size in filled SSBR/BR vulcanizates after annealing for different time.

**Fig. 6:**
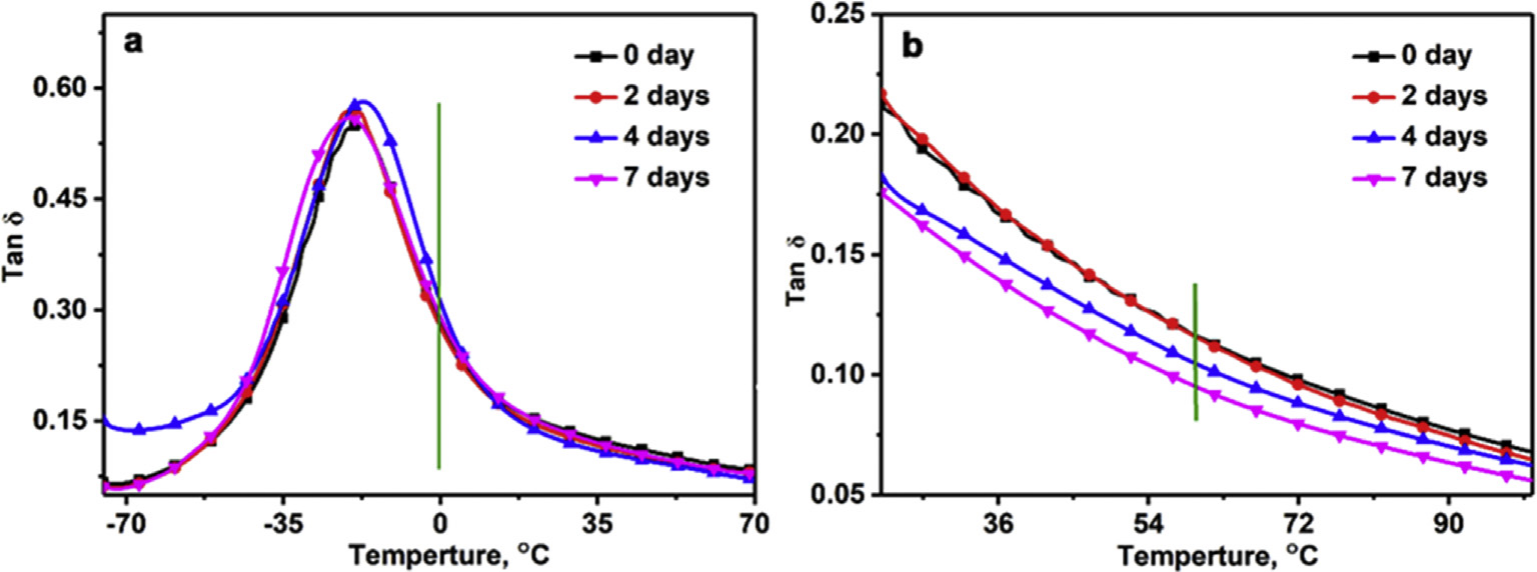
The dynamic mechanical analysis of filled rubber vulcanizates after different annealing periods: a) frequency: 10 Hz, strain: 0.1%; b) frequency: 10 Hz, strain: 5%.

## 2 Experimental design, materials, and methods

The prepared SSBR/BR blends and SSBR/BR/SiO_2_ composites were annealed strictly at room temperature for 0 day, 2 days, 4 days, and 7 days at 23 ± 0.1 °C. The experimental design, materials and methods were performed following our recent reports [1].

### 2.1 Specimen preparation

The un-vulcanized SSBR/BR blends and SSBR/BR/SiO_2_ composites after annealing for 0 day, 2 days, 4 days, and 7 days were made into dumbbell-shaped specimen (Fig. 1) for green strength test. Materials after corresponding annealing time are used for the investigation of crosslinking characteristics. Filler dispersion, and dynamic mechanical properties of rubber vulcanizates are measured after vulcanization at 150 °C under 10 MPa.

### 2.2 Green strength of un-vulcanized rubber gums and compounds

The green strengths of un-vulcanized SSBR/BR gums and SSBR/BR/SiO_2_ compounds which were annealed at room temperature for 0 day, 2 days, 4 days, and 7 days were tested at a speed of 100 mm/min at room temperature by Zwick/Roll electrical tensile tester. The data was plotted into Fig. 2.

### 2.3 Crosslinking characteristics analysis

After the rubber materials were annealed at room temperature for different periods, the vulcanization characteristics of the rubber materials were obtained by a rotor-free vulcameter for 30 minutes (unfilled SSBR/BR gums) and 45 minutes (filled SSBR/BR compounds) at 877 psi, a strain of 7% and a frequency of 1.67 Hz.

### 2.4 Macroscopic filler dispersion analysis

The annealed filled SSBR/BR/SiO_2_ sample were cured at 150 °C and then cut into a test piece with a cross-section of approximately 8 mm in thickness and 10 mm in width. The macroscopic filler dispersion of the filled vulcanizates were examined using a disper GRADER αView filler disperser according to the ISO 11345-2006. The filler dispersion image and size distribution of the filler aggregates in the rubber matrix was obtained after computer fitting.

### 2.5 Dynamic mechanical properties analysis

DMA dynamic mechanical analyzer (Q800, TA instruments) was utilized to investigate the dynamic mechanical properties of SSBR/BR/SiO_2_ vulcanizates with different annealing periods. Each sample was 10 mm in length, 4 mm in width, and 2 mm in thickness. Testing conditions for obtaining the value of tan δ at 0 °C were as follows: temperature sweep was conducted at a frequency of 10 Hz from −80 °C to 100 °C with a heating rate of 3 °C/min and a strain amplitude of 0.1%. Testing conditions for obtaining the value of tan δ at 60 °C were as follows: temperature sweep was conducted at a frequency of 10 Hz from 20 °C to 100 °C with a heating rate of 3 °C/min and a strain amplitude of 5.0%.
