# Supplementary material for: Data on the evolution of curing characteristics and properties during the room-temperature annealing process in SSBR/BR gums and SSBR/BR/SiO2 composites
Source: Data Brief. 2019 Oct 16;27:104660. doi: 10.1016/j.dib.2019.104660 (PMC6833471; doi:10.1016/j.dib.2019.104660)

Quick report

Sample information

Batch	0	Compound	20190530
Order	cl		

Instrument information

Instrument type:	Dispergrader
Instrument name:	disperGRADER av 660AAG1210
Instrument model:	disperGRADERav
Instrument serial no.:	

LYL-G_RCB_Silica : Completed

Start time : 5/30/2019 10:04:34 AM End time : 5/30/2019 10:05:51 AM

Setpoints

Color channel for analysis	Blue	%	Reference bank	G_RCB_Silica	[ ]
Threshold for dispersion calculation	23	um	Volume fraction of the filler	30	%
Nodge to agglomerate fraction	1	[ ]	Exposure time	12	ms
White area threshold	0.0	um			

Datapoints

Agglomerate size std. dev.	6	um	Dispersion	99.1	%
White area	2.0	%	X	10.0	[ ]
Y	9.9	[ ]	Z	94.4	%
Mean agglomerate size	11.1	um			

Images

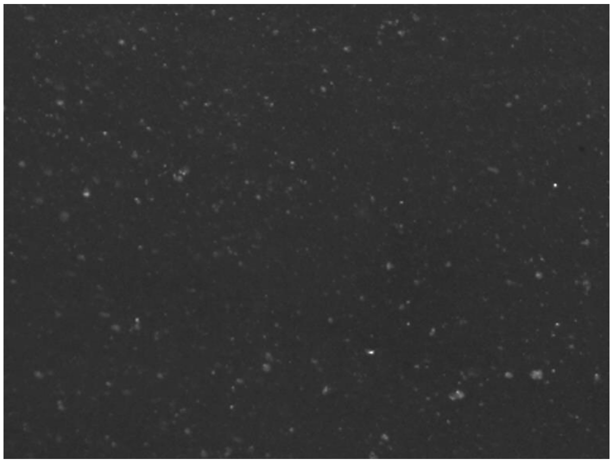

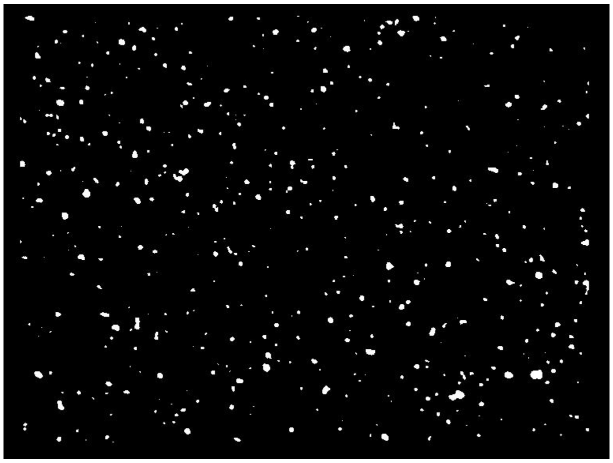

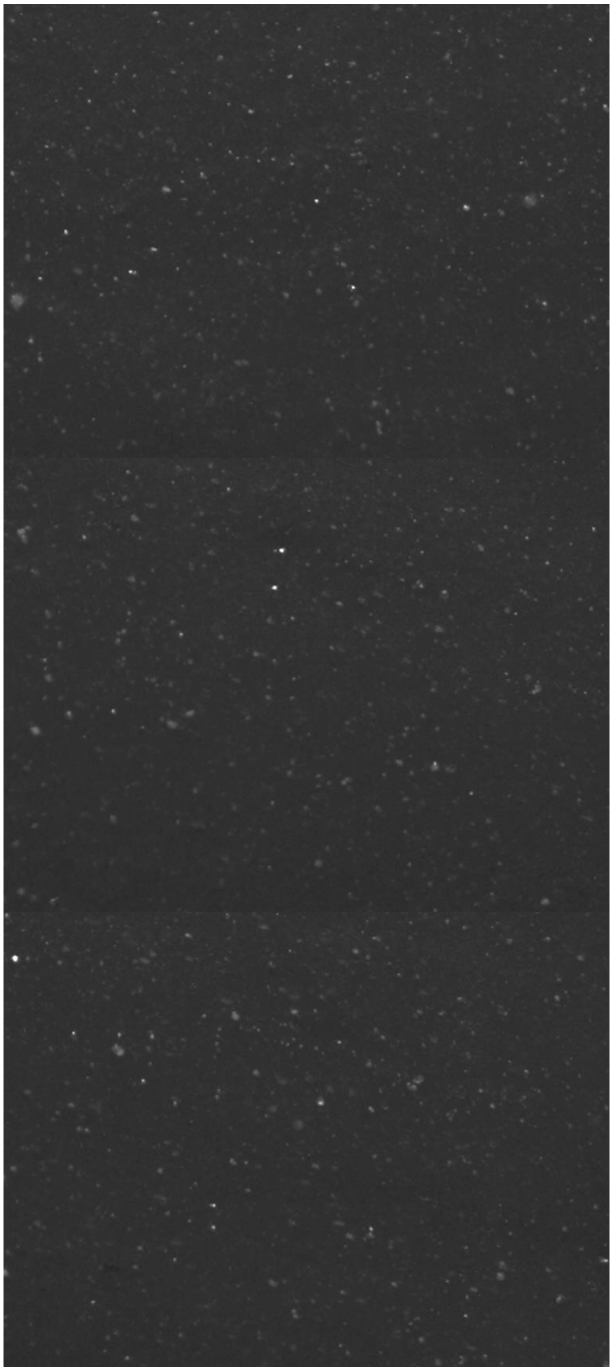

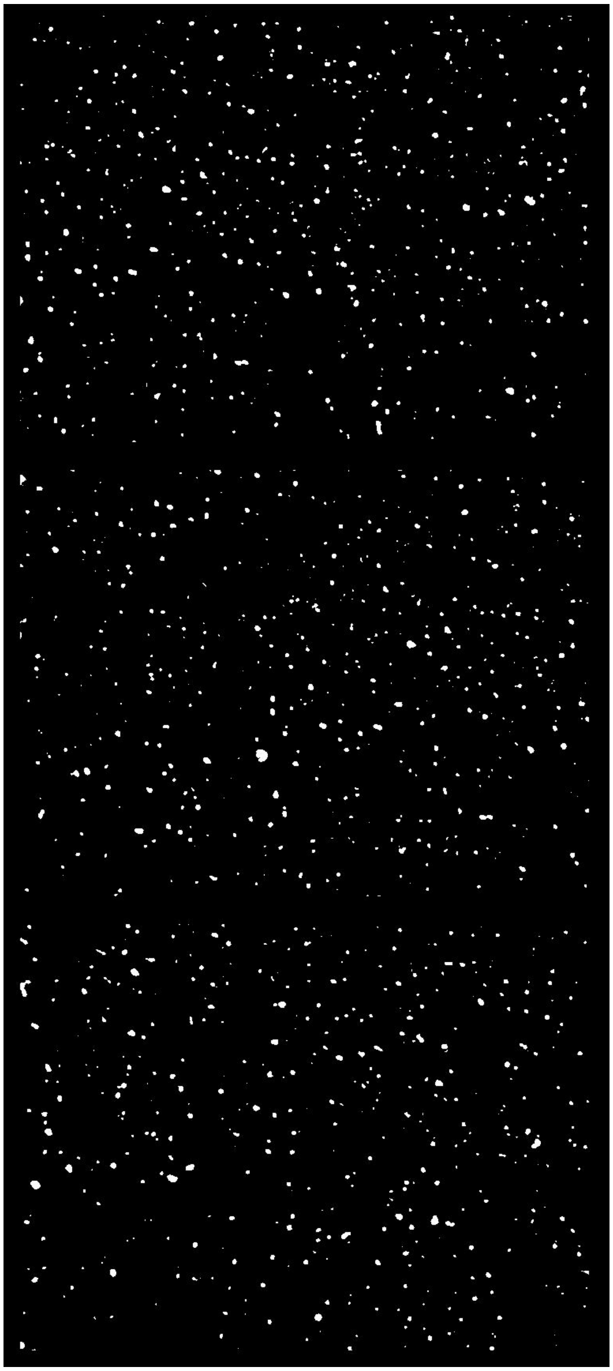

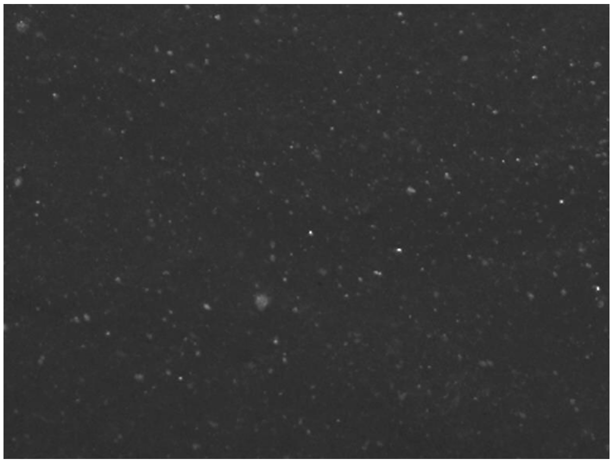

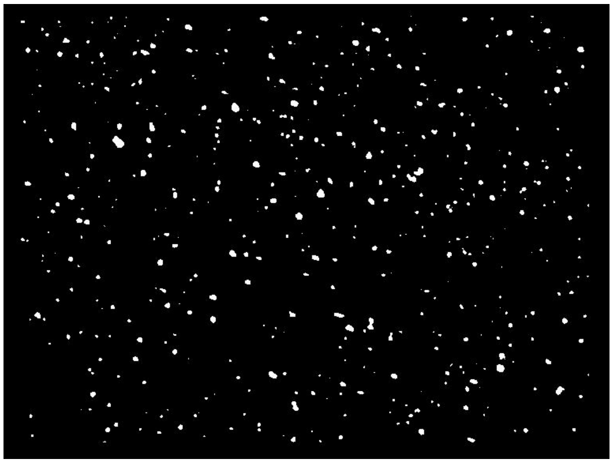

Profiles

Results Top

Spot ID	X	Y	Z [%]	Mean agglomerate size [um]	Agglomerate size std. dev. [um]	Dispersion [%]	White area [%]
1	10.00	9.85	94.66	11.53	6.27	98.93	1.87
2	10.00	9.90	94.73	10.64	5.93	99.33	1.85
3	10.00	9.80	94.01	11.44	6.52	98.59	2.10
4	10.00	9.87	94.22	10.97	6.21	99.10	2.02
5	10.00	9.92	94.32	10.97	5.71	99.45	1.99
Overall	10.00	9.87	94.39	11.11	6.13	99.08	1.96

Histograms

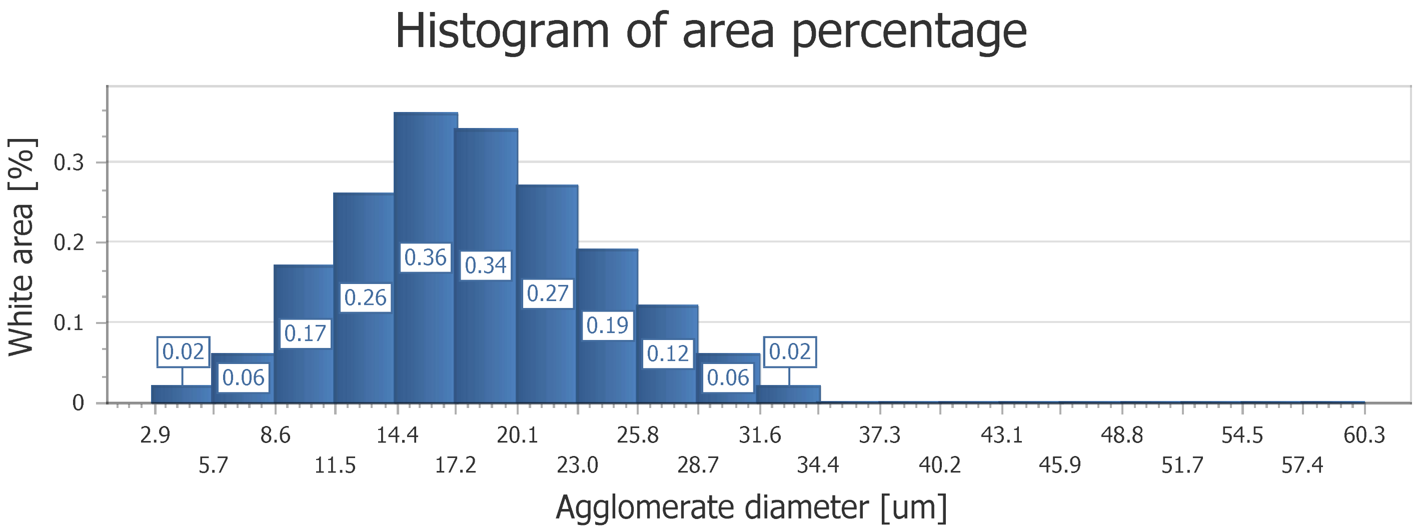

Histogram of agglomerates

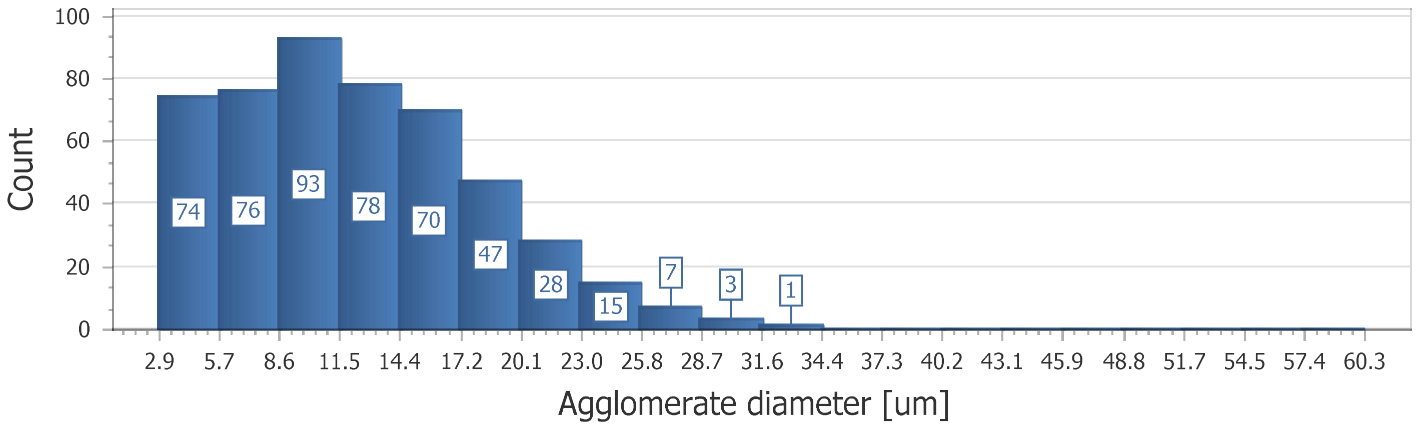

Supplement: Multimedia component 1 [file mmc1.zip › Raw data/Macroscopic filler dispersion.zip!/Macroscopic filler dispersion/0 day.pdf]
