# Supplementary material for: Data on the evolution of curing characteristics and properties during the room-temperature annealing process in SSBR/BR gums and SSBR/BR/SiO2 composites
Source: Data Brief. 2019 Oct 16;27:104660. doi: 10.1016/j.dib.2019.104660 (PMC6833471; doi:10.1016/j.dib.2019.104660)

Quick report

Sample information

Batch	4t	Compound	20190530
Order	cl		

Instrument information

Instrument type:	Dispergrader
Instrument name:	disperGRADER av 660AAG1210
Instrument model:	disperGRADERav
Instrument serial no.:	

LYL-G_RCB_Silica : Completed

Start time : 5/30/2019 9:53:23 AM End time : 5/30/2019 9:55:19 AM

Setpoints

Color channel for analysis	Blue	%
Threshold for dispersion calculation	23	um
Nodge to agglomerate fraction	1	[ ]
White area threshold	0.0	um

Reference bank	G_RCB_Silica	[ ]
Volume fraction of the filler	30	%
Exposure time	12	ms

Datapoints

Agglomerate size std. dev.	7	um
White area	1.6	%
Y	9.8	[ ]
Mean agglomerate size	12.6	um

Dispersion	98.5	%
X	10.0	[ ]
Z	95.3	%

Images

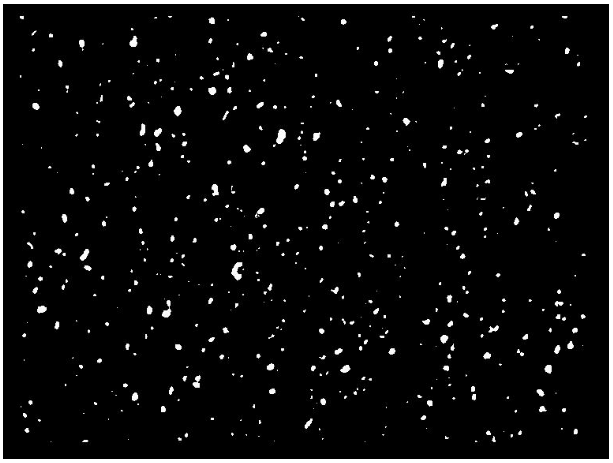

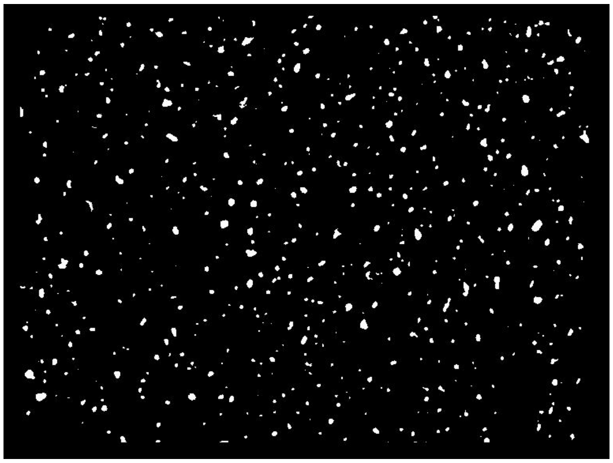

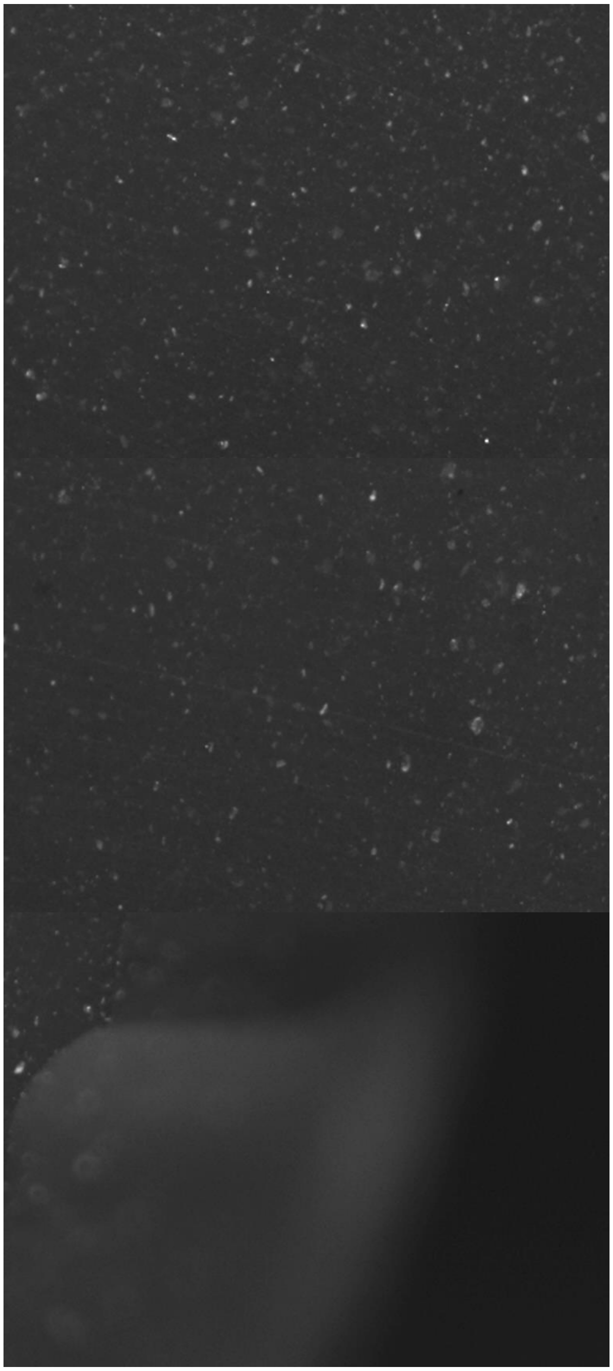

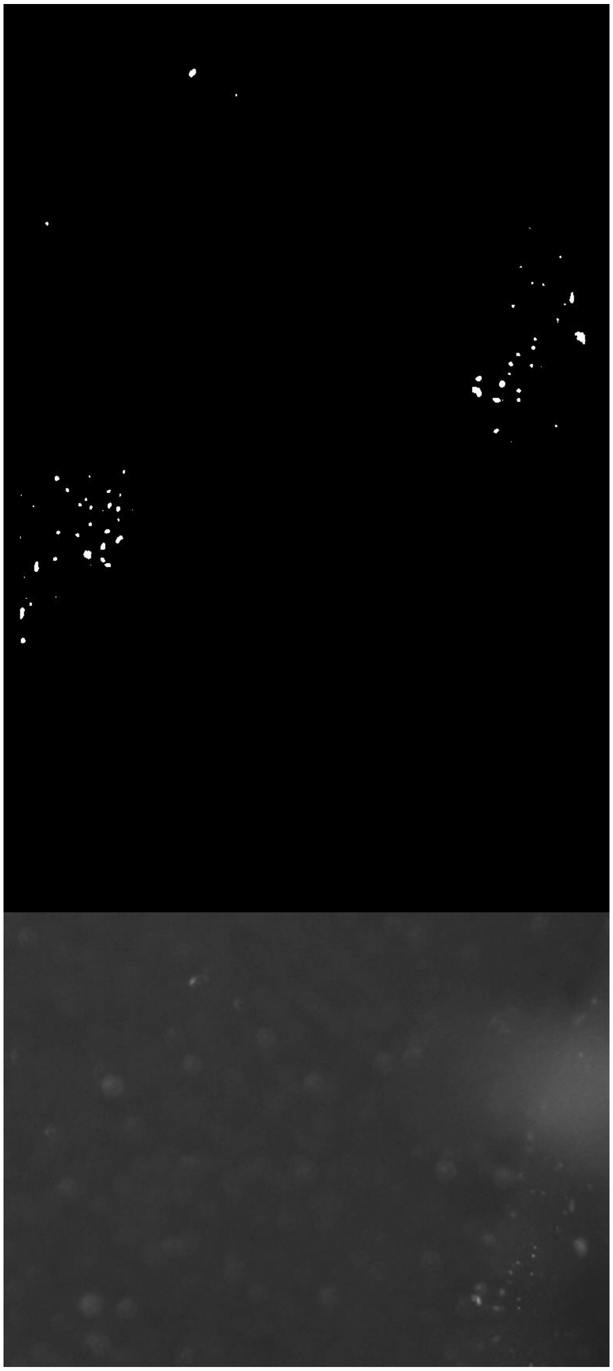

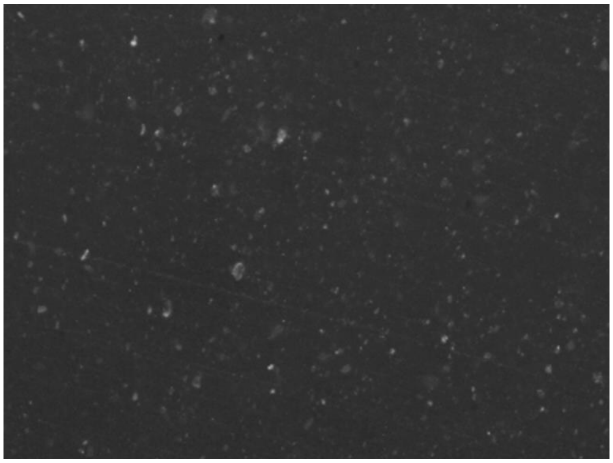

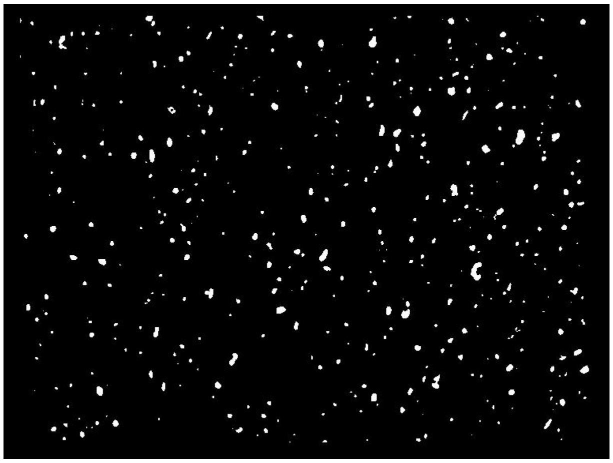

Profiles

Results Top

Spot ID	X	Y	Z [%]	Mean agglomerate size [um]	Agglomerate size std. dev. [um]	Dispersion [%]	White area [%]
1	10.00	9.64	91.01	12.42	6.77	97.47	3.15
2	10.00	9.97	99.47	12.80	7.41	99.82	0.19
3	10.00	9.68	93.05	11.79	7.11	97.76	2.43
4	10.00	9.71	93.50	12.25	7.05	97.95	2.27
5	10.00	9.95	99.44	13.51	8.90	99.68	0.20
Overall	10.00	9.79	95.29	12.55	7.45	98.54	1.65

Histograms

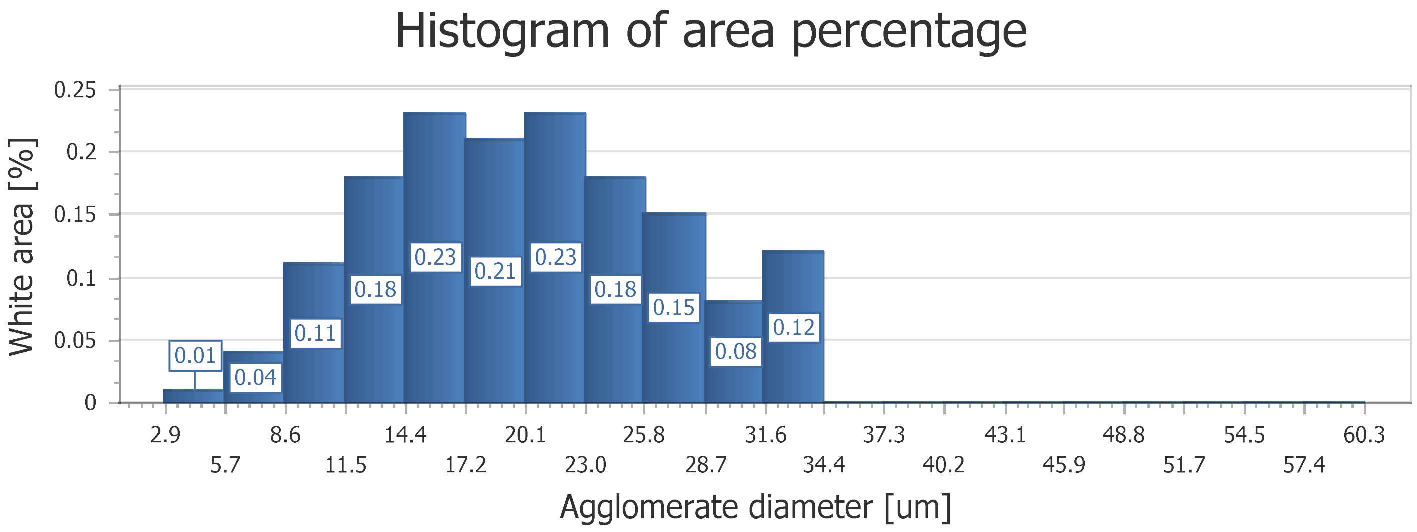

Histogram of agglomerates

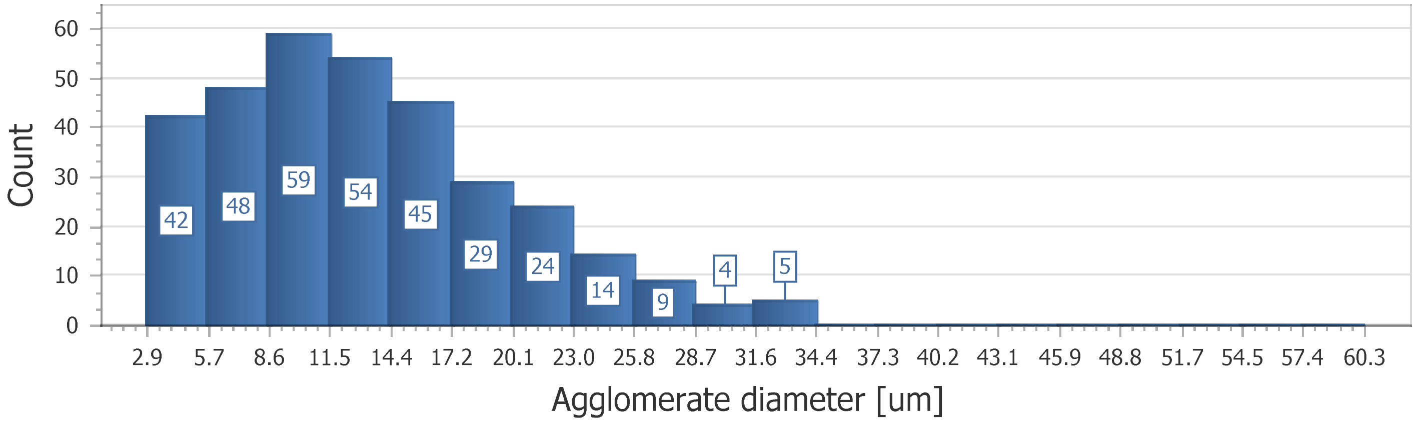

Supplement: Multimedia component 1 [file mmc1.zip › Raw data/Macroscopic filler dispersion.zip!/Macroscopic filler dispersion/2 days.pdf]
