# Supplementary material for: Data on the evolution of curing characteristics and properties during the room-temperature annealing process in SSBR/BR gums and SSBR/BR/SiO2 composites
Source: Data Brief. 2019 Oct 16;27:104660. doi: 10.1016/j.dib.2019.104660 (PMC6833471; doi:10.1016/j.dib.2019.104660)

Quick report

Sample information

Batch	0t3	Compound	20190530
Order	cl		

Instrument information

Instrument type:	Dispergrader
Instrument name:	disperGRADER av 660AAG1210
Instrument model:	disperGRADERav
Instrument serial no.:	

LYL-G_RCB_Silica : Completed

Start time : 5/30/2019 10:02:35 AM End time : 5/30/2019 10:03:44 AM

Setpoints

Color channel for analysis	Blue	%	Reference bank	G_RCB_Silica	[ ]
Threshold for dispersion calculation	23	um	Volume fraction of the filler	30	%
Nodge to agglomerate fraction	1	[ ]	Exposure time	12	ms
White area threshold	0.0	um			

Datapoints

Agglomerate size std. dev.	7	um	Dispersion	98.2	%
White area	2.3	%	X	10.0	[ ]
Y	9.7	[ ]	Z	93.3	%
Mean agglomerate size	11.8	um			

Images

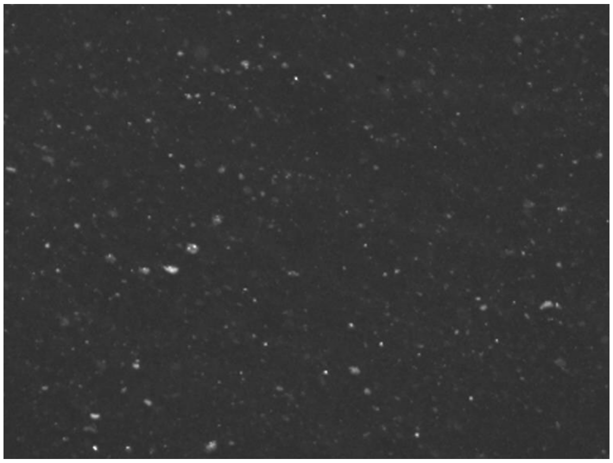

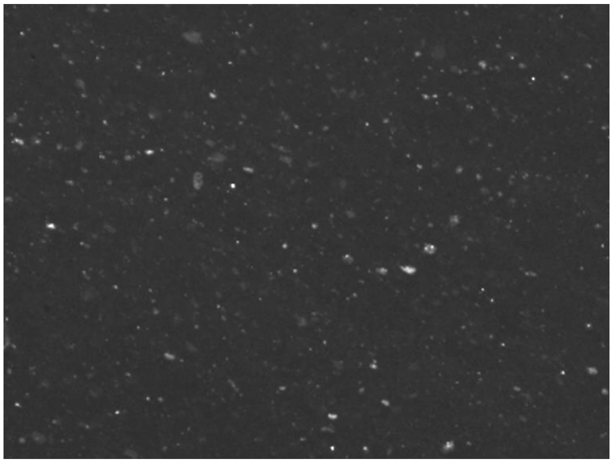

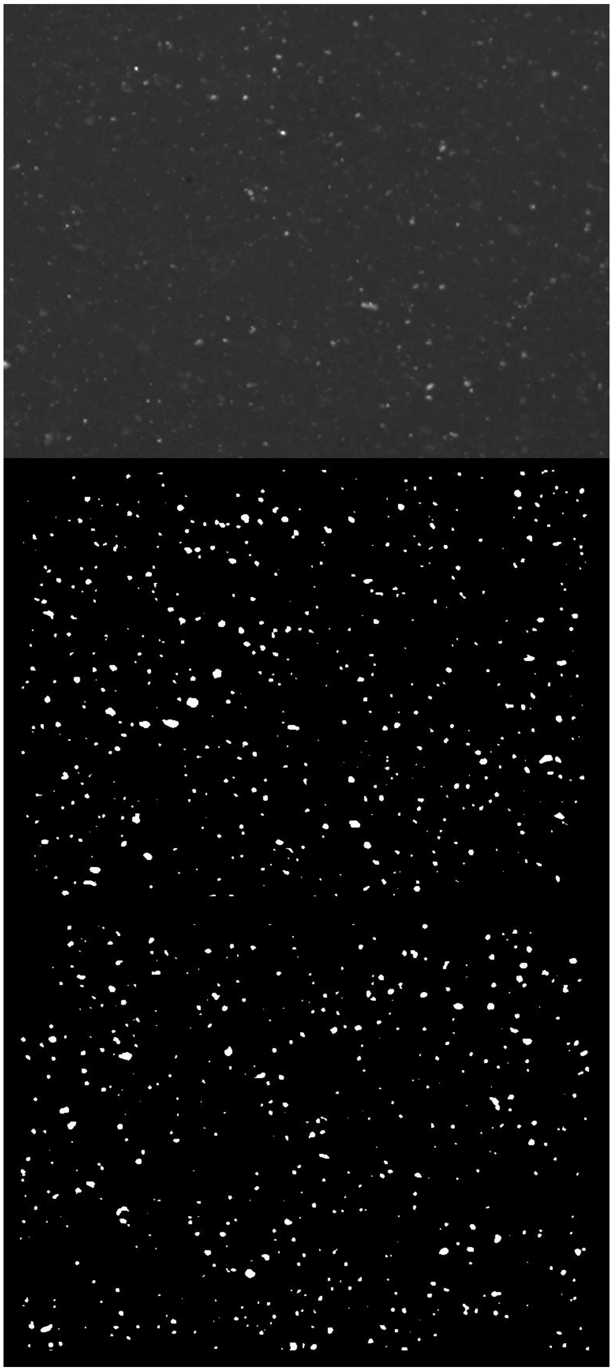

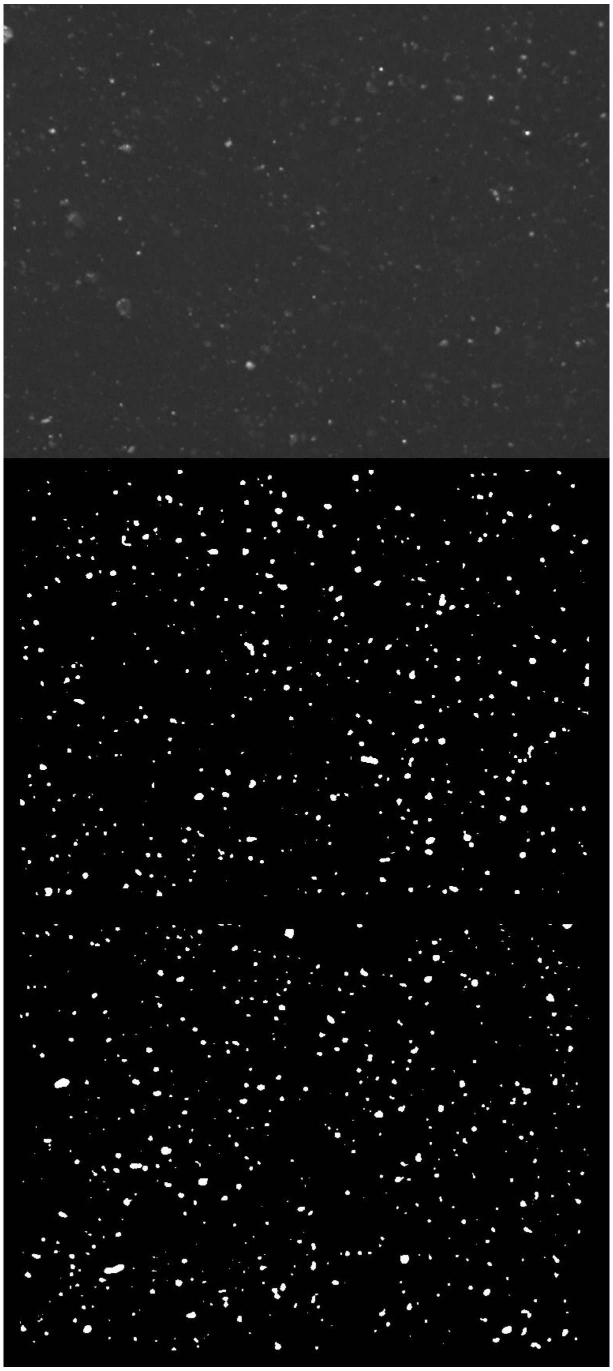

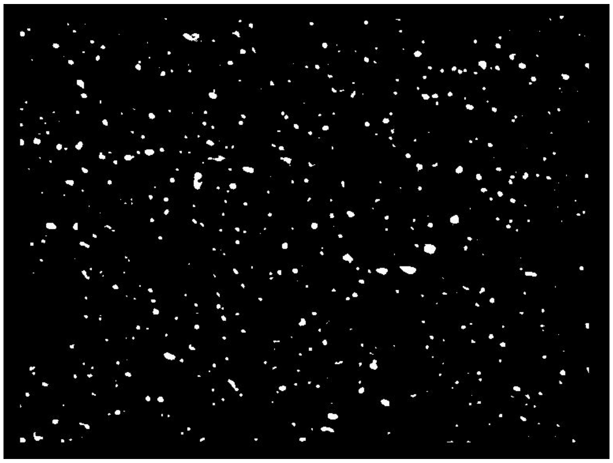

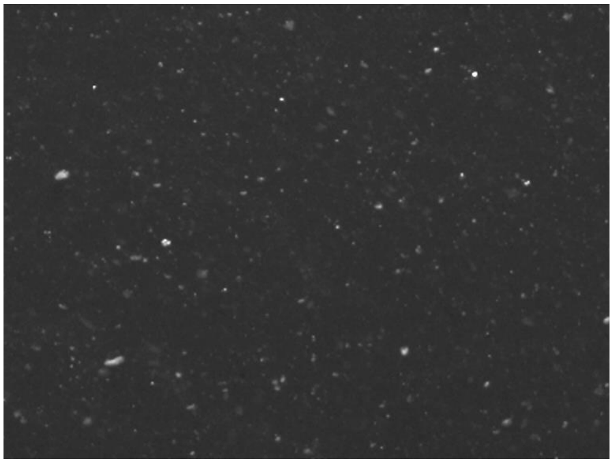

Profiles

Results Top

Spot ID	X	Y	Z [%]	Mean agglomerate size [um]	Agglomerate size std. dev. [um]	Dispersion [%]	White area [%]
1	10.00	9.70	93.05	11.34	7.08	97.91	2.43
2	10.00	9.80	93.84	11.96	6.66	98.58	2.16
3	10.00	9.76	93.43	11.73	6.66	98.31	2.30
4	10.00	9.71	93.04	11.73	6.92	97.95	2.44
5	10.00	9.76	93.22	12.24	6.62	98.34	2.37
Overall	10.00	9.75	93.32	11.80	6.79	98.22	2.34

Histograms

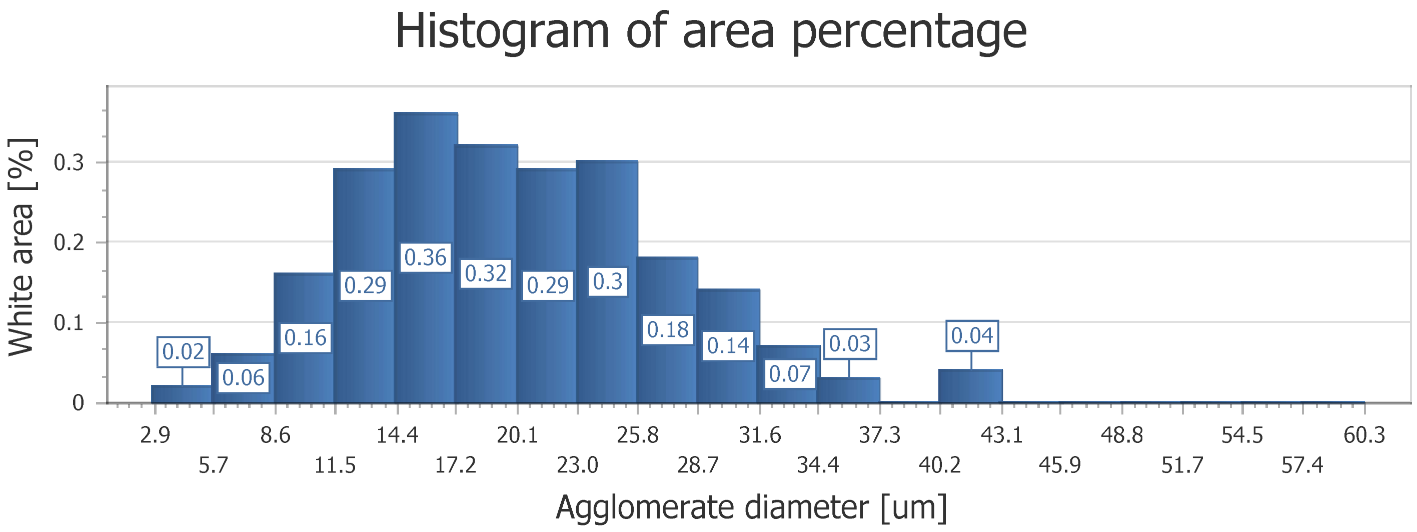

Histogram of agglomerates

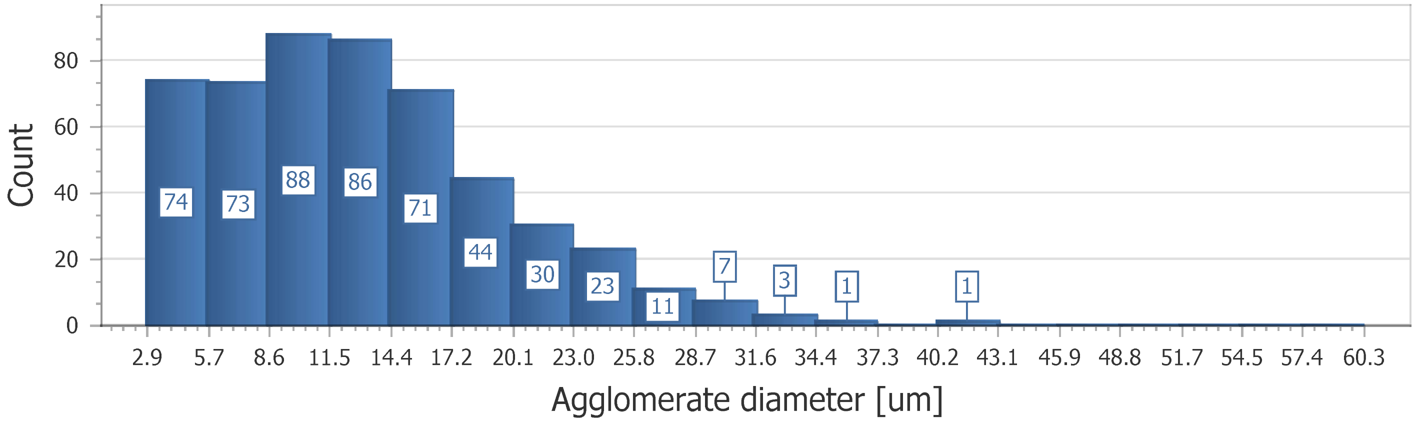

Supplement: Multimedia component 1 [file mmc1.zip › Raw data/Macroscopic filler dispersion.zip!/Macroscopic filler dispersion/4 days.pdf]
