# Supplementary material for: Data on the evolution of curing characteristics and properties during the room-temperature annealing process in SSBR/BR gums and SSBR/BR/SiO2 composites
Source: Data Brief. 2019 Oct 16;27:104660. doi: 10.1016/j.dib.2019.104660 (PMC6833471; doi:10.1016/j.dib.2019.104660)

Quick report

Sample information

Batch	0t2	Compound	20190530
Order	cl		

Instrument information

Instrument type:	Dispergrader
Instrument name:	disperGRADER av 660AAG1210
Instrument model:	disperGRADERav
Instrument serial no.:	

LYL-G_RCB_Silica : Completed

Start time : 5/30/2019 10:00:11 AM End time : 5/30/2019 10:01:17 AM

Setpoints

Color channel for analysis	Blue	%	Reference bank	G_RCB_Silica	[ ]
Threshold for dispersion calculation	23	um	Volume fraction of the filler	30	%
Nodge to agglomerate fraction	1	[ ]	Exposure time	12	ms
White area threshold	0.0	um			

Datapoints

Agglomerate size std. dev.	7	um	Dispersion	97.6	%
White area	2.8	%	X	10.0	[ ]
Y	9.7	[ ]	Z	91.9	%
Mean agglomerate size	11.8	um			

Images

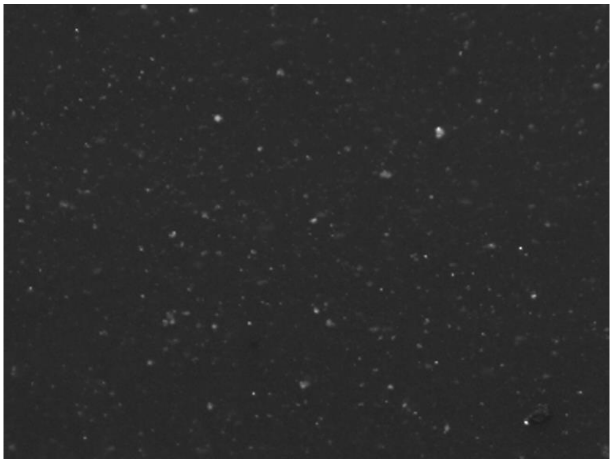

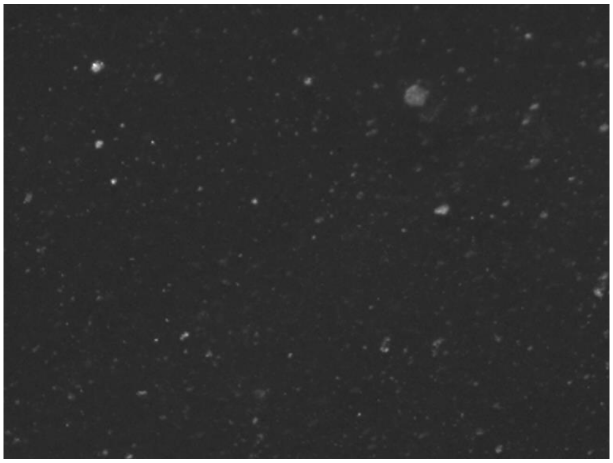

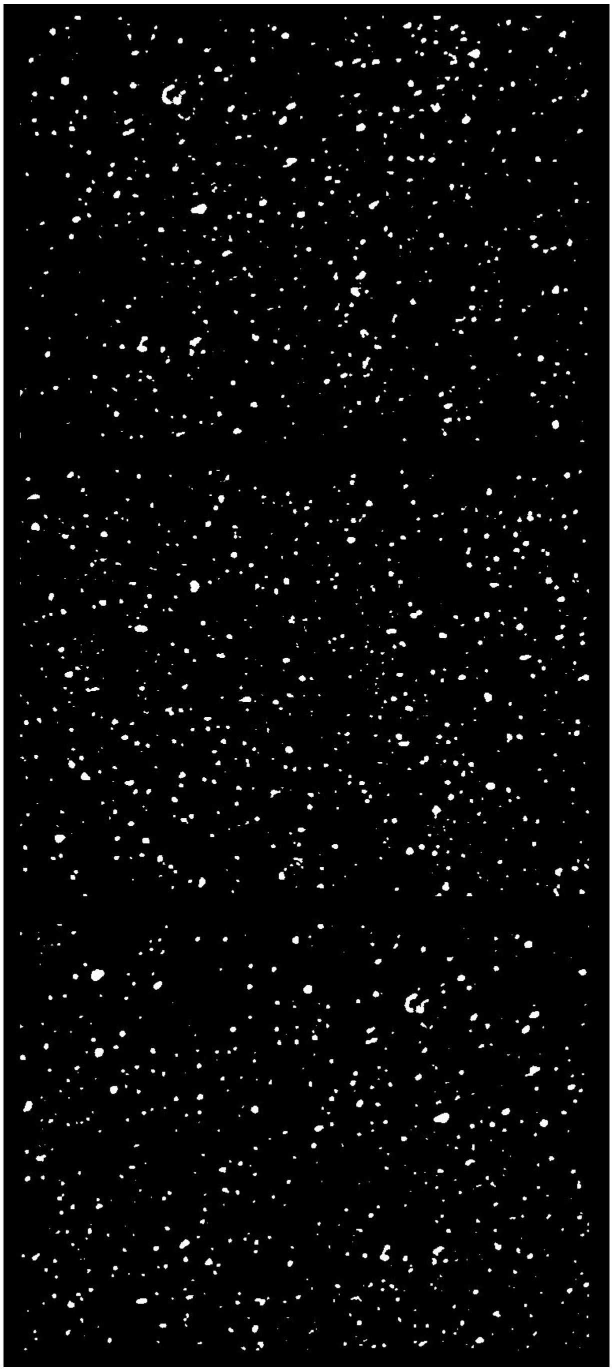

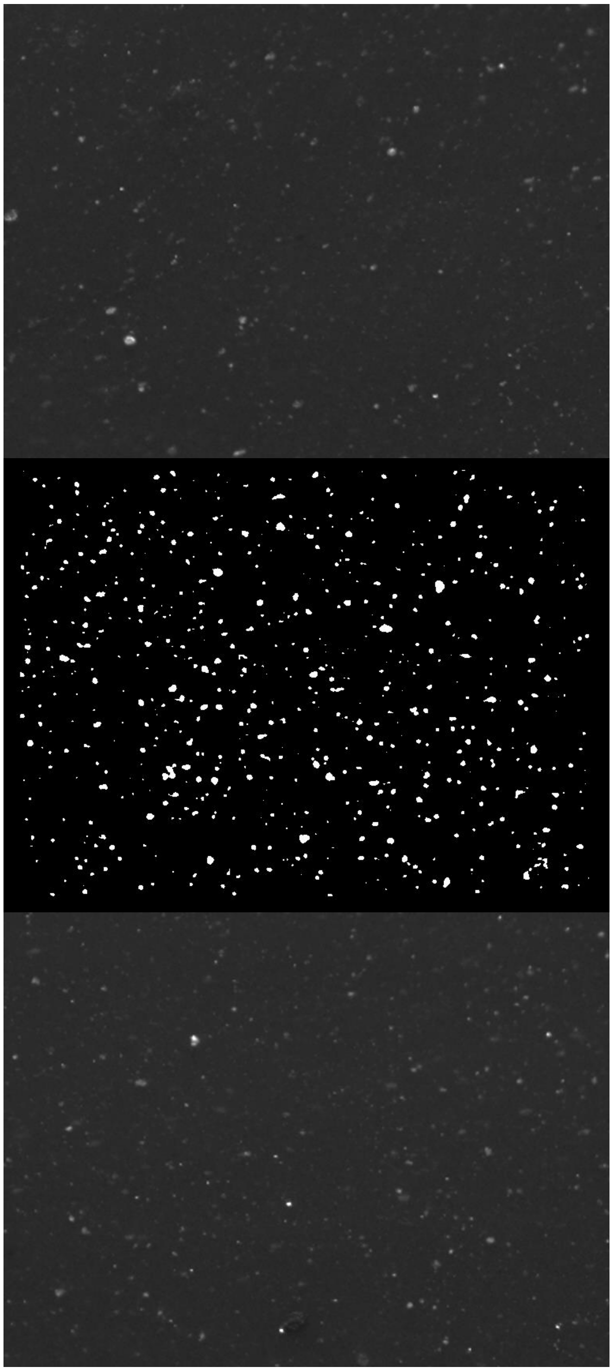

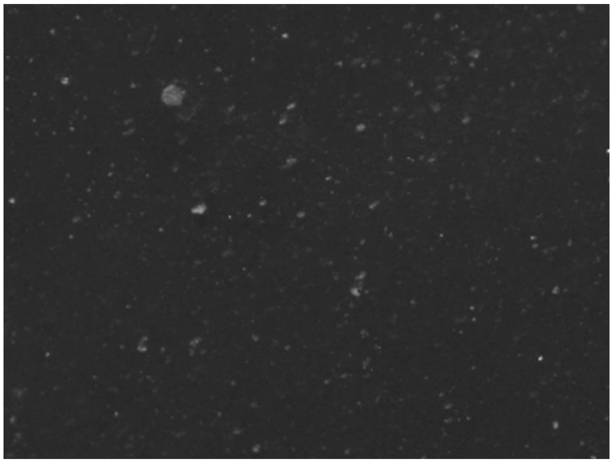

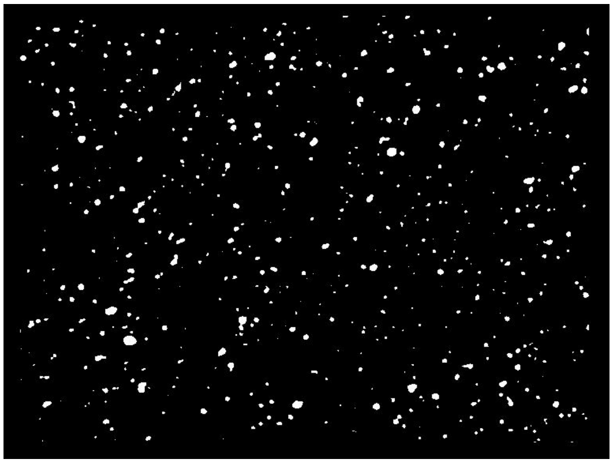

Profiles

Results Top

Spot ID	X	Y	Z [%]	Mean agglomerate size [um]	Agglomerate size std. dev. [um]	Dispersion [%]	White area [%]
1	10.00	9.67	92.44	11.41	6.93	97.70	2.65
2	10.00	9.71	91.33	11.86	6.72	97.94	3.03
3	10.00	9.63	91.90	12.24	7.20	97.41	2.84
4	10.00	9.61	92.42	11.86	7.21	97.28	2.65
5	9.91	9.65	91.22	11.87	6.73	97.54	3.07
Overall	9.98	9.66	91.86	11.85	6.96	97.57	2.85

Histograms

Histogram of area percentage

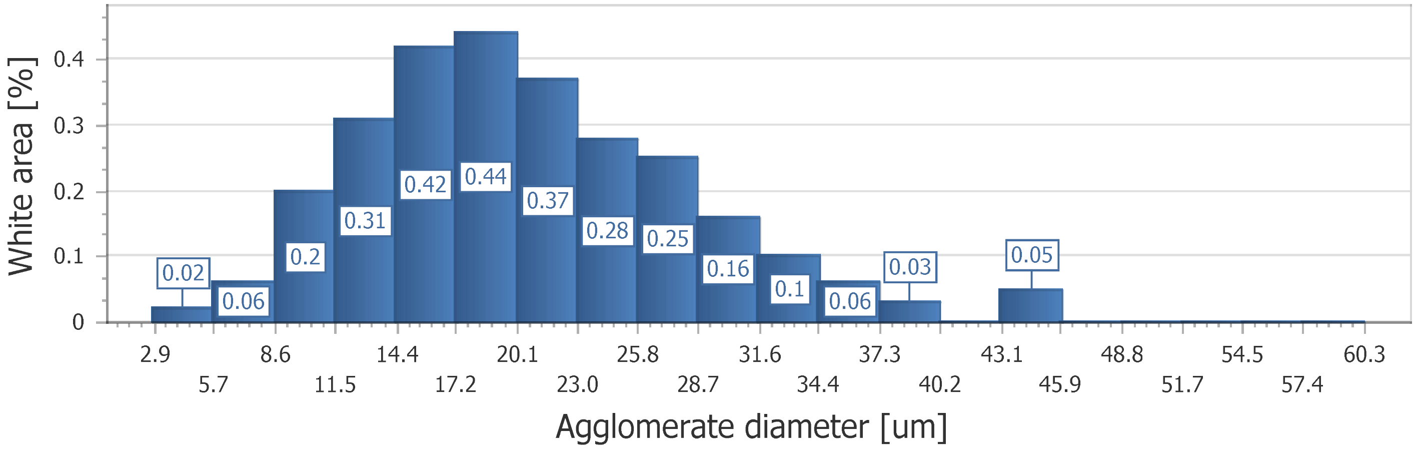

Histogram of agglomerates

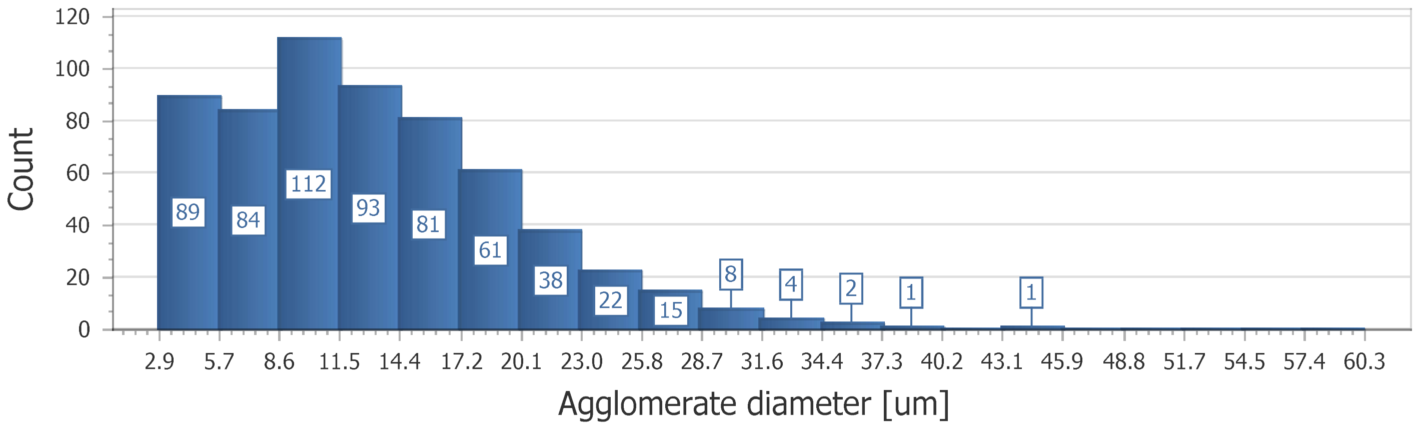

Supplement: Multimedia component 1 [file mmc1.zip › Raw data/Macroscopic filler dispersion.zip!/Macroscopic filler dispersion/7 days.pdf]
